# Paving the way ahead: protocol optimization of mouse models in crush syndrome related acute kidney injury research

**DOI:** 10.3389/fphar.2024.1438127

**Published:** 2024-09-30

**Authors:** Ou Qiao, Xinyue Wang, Zizheng Li, Lu Han, Xin Chen, Li Zhang, Fengjiao Bao, Herui Hao, Yingjie Hou, Xiaohong Duan, Sania Saeed, Ning Li, Yanhua Gong

**Affiliations:** ^1^ School of Disaster and Emergency Medicine, Faculty of Medicine, Tianjin University, Tianjin, China; ^2^ Institute of Disaster and Emergency Medicine, Faculty of Medicine, Tianjin University, Tianjin, China; ^3^ Medical School, Faculty of Medicine, Tianjin University, Tianjin, China; ^4^ Key Laboratory for Disaster Medicine Technology, Tianjin, China

**Keywords:** crush syndrome, acute kidney injury, mouse model, GFR, rhabdomyolysis

## Abstract

**Backgrounds:**

Crush syndrome (CS) is the leading cause of death after earthquakes, second only to direct trauma. Acute kidney injury (AKI) is the most severe complication of CS. Research based on the CS-AKI mouse model and kidney function assessment by glomerular filtration rate (GFR) helps to elucidate the pathogenesis of CS-AKI, which contributes to effective treatment measures.

**Methods:**

Mice were modeled by the multi-channel small animal crushing platform. We set up different CS-AKI modeling parameters by applying different crushing weights (0.5 kg, 1.0 kg, 1.5 kg), crushing durations (6 h, 12 h, 16 h), and decompression durations (6 h, 12 h, 24 h). The GFR, serum creatinine (SCr), blood urea nitrogen (BUN), kidney tissue *Kim-1* mRNA and *Ngal* mRNA expression levels, and HE staining were examined to evaluate the results of different protocols.

**Results:**

The results showed that with the crushing weight increased, the kidney function assessment’s gold standard GFR significantly decreased, and the levels of SCr and BUN increased. Meanwhile, the longer crushing durations found a higher extension of inflammatory cell infiltration in the kidney. The degree of kidney injury continued to worsen with the duration of decompression, indicating severe damage after reperfusion, which was associated with tubular injury and a sustained elevation of the inflammatory state.

**Conclusion:**

We successfully constructed CS-AKI mouse models with different severities under the above parameters. Applying 1.5 kg for 16 h and then decompressing for 24 h induced severe AKI. These findings provide clues for further exploration of the mechanism and treatment of traumatic AKI.

## Introduction

Crush syndrome (CS), also known as traumatic rhabdomyolysis (RM) syndrome, occurs when the body’s muscles and areas with a rich blood supply are continuously crushed by external pressure (Li et al., 2022). The crushed area suffers from ischemia-reperfusion injury after the crushing weight is relieved and ultimately leads to RM, which is a clinically critical illness characterized mainly by systemic circulatory failure and acute kidney injury (Qiao et al., 2023). Since Bywaters, etc., formally introduced this concept during the London Blitz in 1940, CS has gradually been recognized as an important cause of death in earthquakes, tsunamis, wars, and major traffic accidents (Better, 1997). CS is the leading cause of indirect death after earthquakes. It was reported that 237 of 639 patients were diagnosed with CS, and 41 of them died at Kayseri State Hospital after a 7.8 magnitude earthquake in Turkey in 2023 (Koyuncu et al., 2023). CS has many complications with high mortality rates and poor prognoses, and the kidney is the primary target organ involved (Ramirez-Guerrero et al., 2024). Acute kidney injury (AKI) is the most severe complication and a lethal factor in CS (Ramirez-Guerrero et al., 2024). At present, the treatment of CS-AKI mainly focuses on liquid therapy, urine alkalization, and diuretic support to improve renal tubular blood flow and pH value. It also includes renal replacement therapy to remove circulating myoglobin. However, due to limitations in the amount of equipment and medical personnel at a disaster site, it may not benefit most patients in time (Vanholder et al., 2010; Forni et al., 2024). Therefore, it is urgent to explore the pathological mechanisms of CS to develop new treatments and improve clinical management.

Research based on animal models is significant for early diagnosis and treatment of CS, and it may improve the prognosis of patients. Currently, research on CS animals is mainly aimed at exploring the pathological mechanism of CS, searching for specific disease markers, and finding new treatment methods. The model animals for CS include rats (68.3%), mice (13.4%), rabbits (11.0%), canines (4.9%), pigs, and goats (Luo et al., 2023). The construction methods of the CS models include non-invasive physical crush injuries (such as heavy objects (Song et al., 2015), rubber tourniquets (Murata et al., 2020), glycerin muscle injection-induced RM (Okubo et al., 2018), and myoglobin (Mb) injection (Heyman et al., 1996). These model animal studies are designed to reflect the clinical characteristics of CS better. However, from the published articles, the intensity of the crushing weight, the crushing area, the stability of the crushing device, and the evaluation indicators for the model will directly affect the judgment of the results and the repeatability of the experiments. The main reason is the lack of suitable modeling processes and evaluation standards. A research system suitable for the rat CS model has been developed, which will make an outstanding contribution to this field and provide a valuable reference for future research (Li et al., 2023). However, a research system on mice has not yet been established. With rapid development, ease of government, and low-cost reproduction and maintenance, laboratory mice have contributed significantly to scientific research on many diseases (Israelow et al., 2020). In addition, mice are more suitable for immunological research and are often the first choice of model animals for evaluating new vaccines (Fiege et al., 2021). We also note that in recent years, scholars worldwide, including our research team, have begun immunotherapy attempts for CS, which will undoubtedly open up new fields for the early treatment of CS (Shimazaki et al., 2012; Wang et al., 2023). Therefore, establishing a suitable mouse model and evaluation standards for CS will lay the foundation for the following immunotherapy, drug pharmacology, and toxicology studies.

This study aims to find a suitable CS-AKI mouse model protocol by optimizing the crushing weight, crushing duration, and decompression duration combination with a multi-channel small animal crushing platform. This is combined with evaluating the gold standard of kidney function (GFR), kidney injury biomarkers, and molecular pathology techniques. In addition, we also compared the consistency and differences between the CS-AKI mouse model and the glycerin-induced RM model. We hope to provide insights for the future drug development and immune therapy of CS-AKI.

## Materials and methods

### Animal and CS-AKI mouse models

**FIGURE 1:**
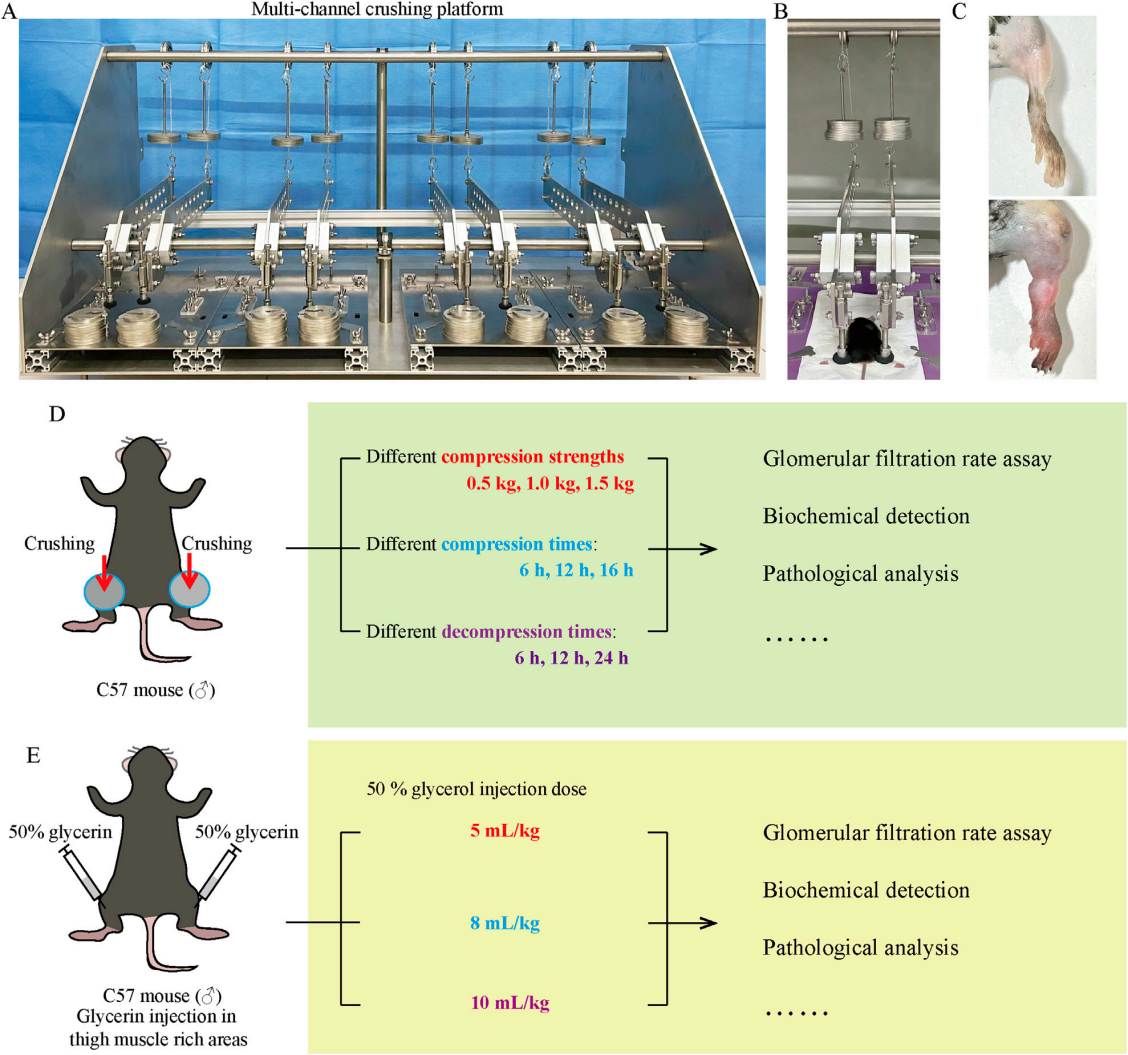
CS model equipment and research schedule. **(A)** Multi-channel small animal crushing platform made by our research team. **(B)** Ongoing CS mouse model establishment. **(C)** Comparison of thighs between NC group and crushed mice. **(D, E)** CS and RM mice study schedule.

The male C57BL/6J mice (8–10 weeks old, 18 ± 22 g) were purchased from SPF (Beijing) Biotechnology Co., Ltd. (using male mice to avoid estrogenic effects). All mice were housed in a specific pathogen-free husbandry with 24°C, 12 h light/dark cycles, and had free access to food and water. The study was performed after the mice were acclimated for 1 week. The mice were anesthetized by urethane (1.5 g/kg, *i.p.*), and then their legs were spread apart and fixed on the crushing platform using medical tape. The crush platform was constructed by our laboratory and has applied for a patent in China National Intellectual Property Administration (Application number: 202,211,078,298.9), which is under review (Figure 1A). Briefly, the platform consists of four separate crushing channels, each containing a mouse immobilizer that allows the mouse to be immobilized with its limbs stretched out naturally. Two compression devices are at the position of each thigh of the mouse, which are connected to levers and weights that amplify the weight of the weights by 4 times for crushing. The crushing force can be changed by adjusting the number and weight of the weights. This study investigated the effect of different crushing weights (0.5 kg, 1 kg, and 1.5 kg), crushing duration (6 h, 12 h, and 16 h), and decompression time (6 h, 12 h, and 24 h) on AKI (Figure 1D). When exploring one variable, other factors were kept consistent. For the crushing weight test: fixed crushing duration of 12 h, decompression time of 6 h. Mice were randomly divided into the following four groups: negative control (NC) group, CS_12 h_6 h_0.5 kg group, CS_12 h_6 h_1 kg group, and CS_12 h_6 h_1.5 kg group. For the crushing duration test: fixed crushing weight of 1.5 kg, decompression time of 6 h. Mice were randomly divided into the following four groups: NC group, CS_1.5 kg_6 h_6 h group, CS_1.5 kg_6 h_12 h group, and CS_1.5 kg_6 h_16 h group. For the decompression time test: fixed crushing weight of 1.5 kg, crushing duration of 16 h. Mice were randomly divided into the following four groups: NC group, CS_1.5 kg_16 h_6 h group, CS_1.5 kg_16 h_12 h group, and CS_1.5 kg_16 h_24 h group. After the experiment, the mice were sacrificed, and the serum and kidney tissues were collected for subsequent experiments. The NC group did not perform crush. There were six mice in each group. Animal experiments were performed in line with the principles of the Declaration of Helsinki. Approval was granted by the Ethics Committee of Tianjin University (Date 2022–11–07/NO. TJUE-2022–279).

### RM-AKI mouse model

The RM-AKI mouse model was constructed according to the methods published in previous literature (Muratsu et al., 2022). After the mice were anesthetized by urethane, 50% sterile glycerol (diluted in normal saline) was injected into the bilateral thigh muscle-rich area at 5, 8, and 10 mL/kg (administered in equally divided intramuscular hind limb injections) (Figure 1E).

### Noninvasive percutaneous glomerular filtration rate (GFR) assessment measurement

The GFR of mice was measured by percutaneously monitoring the clearance rate of the FITC-labeled sinistrin tracer. After the mice were anesthetized and shaved, a GFR monitor was adhered to the kidney area of the mice using double-sided adhesive patches (MediBeacon, Mannheim, Germany), and the baseline fluorescence signals were collected for 5 min. Then, FITC-sinistrin (7 mg/100 g b.w.) was injected through the tail vein. The device was removed after collecting the signal for 80 min. The individual percutaneous GFR was determined through FITC-sinistrin elimination kinetic curves, which were analyzed with MB Studio.

### Serum creatinine (SCr) and blood urea nitrogen (BUN) measurement

Mouse blood samples were stored at room temperature for 30 min and then centrifuged at 3,000 rpm for 10 min. The supernatant was collected and used to examine the SCr and BUN levels according to the kit instructions (C011-2-1, C013-2-1, Jiancheng, Nanjing, China).

### HE staining

Briefly, mouse kidney tissue was fixed with 4% paraformaldehyde (P1110, Solarbio, Beijing, China), dehydrated, and embedded in paraffin. Then, the tissue was cut into 5 μm thick sections. Paraffin sections were placed in an oven at 60°C for 2 h, and then deparaffinized to hydrate. Staining was sequentially performed in hematoxylin for 2 min, tap water for 1 min, differentiation solution for 30 s, tap water for 1 min, eosin solution for 1 min, and then dehydrated until sealed with a neutral resin. The histopathologic changes in the kidney tissue were observed and photographed under an optical microscope.

### qPCR

Total RNA from the kidney tissues was extracted using the TRIzol method. The purity and concentration of the extracted RNA samples were determined using Nanodrop One. Reverse transcription into cDNA and amplification were performed using the Hifair^®^ II 1st Strand cDNA Synthesis Kit (11141ES60, Yeasen, Shanghai, China). Primers were synthesized by Tsingke (Tsingke Biotechnology Co., Ltd., Beijing, China). The qPCR reaction was programmed according to SGExcel Fast SYBR qPCR mix (B532955, Sangon Biotech, Shanghai, China), with pre-denaturation at 95°C for 3 min, and PCR reaction at 95°C for 5 s and 60°C for 20 s for a total of 40 cycles. GPADH was used as a loading control for the mRNA measurements. The 2^−ΔΔCt^ method was used to calculate relative expression levels. The primers were listed in Table 1.

**TABLE 1:** Primer sequence.

Gene	Species	Primer sequences (5′–3′)	
*Kim-1*	mouse	Forward	CTG​CTG​CTA​CTG​CTC​CTT​GT
		Reverse	GCA​ACC​ACG​CTT​AGA​GAT​GC
*Ngal*	mouse	Forward	ACG​GAC​TAC​AAC​CAG​TTC​GC
		Reverse	GGG​ACA​GCT​CCT​TGG​TTC​TT
*Gapdh*	mouse	Forward	AGG​TCG​GTG​TGA​ACG​GAT​TTG
		Reverse	TGT​AGA​CCA​TGT​AGT​TGA​GGT​CA

### Statistical analysis

Statistical analysis was performed using SPSS 20.0 software. The results were expressed as mean ± standard deviation (SD). Differences were compared using one-way ANOVA, and further analysis between groups was conducted using the LSD method. *p* < 0.05 was considered statistically significant. All experiments were repeated three times.

## Results

### The impact of different crushing weights on CS-AKI mouse kidney function

**FIGURE 2:**
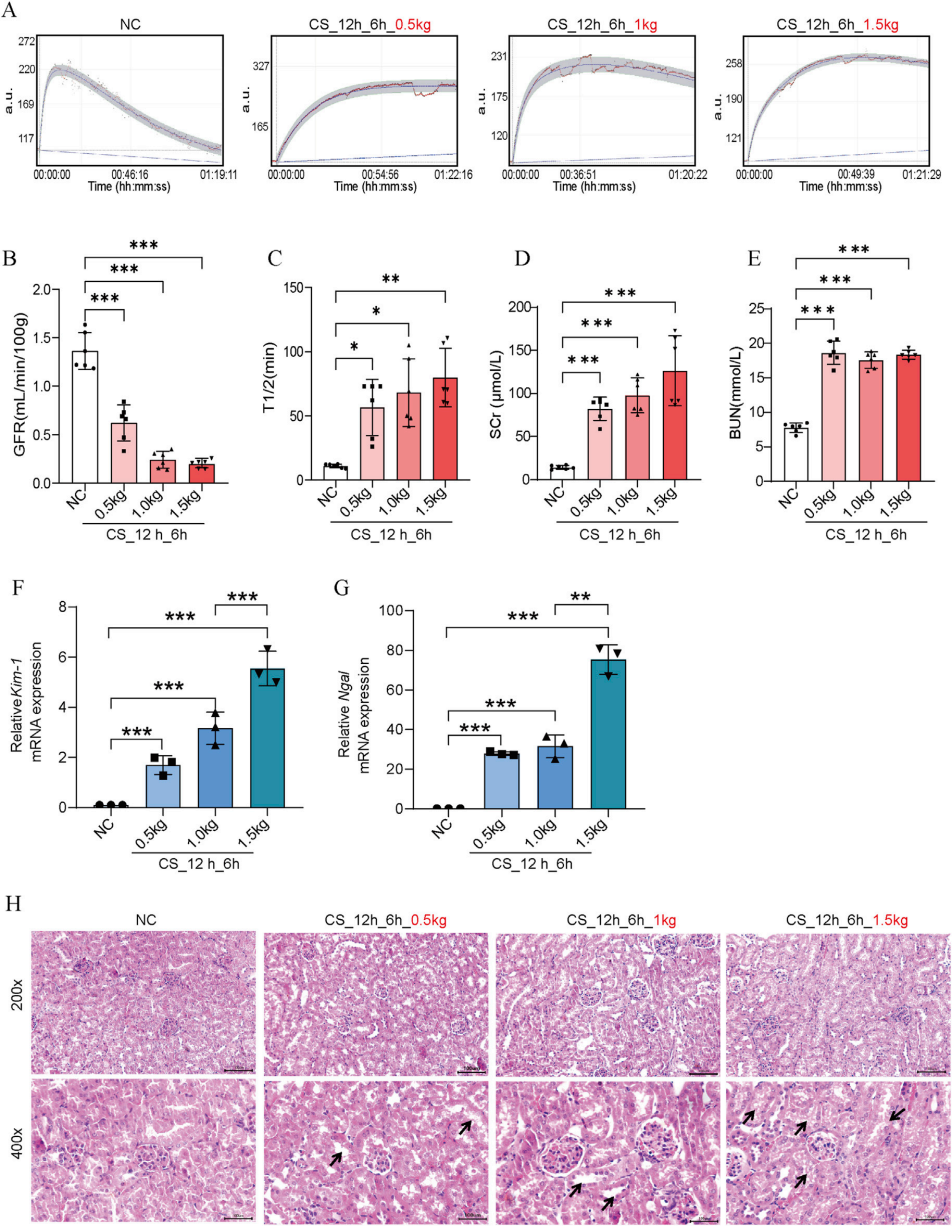
Effects of crush weights on kidney function in CS-AKI mice. For the crushing weight test: fixed crushing duration of 12 h, decompression time of 6 h. The crushing weight applied was 0.5 kg, 1 kg and 1.5 kg respectively. **(A)** GFR of mice subjected to 0.5 kg, 1.0 kg, and 1.5 kg of crushing weight for 12 h and 6 h after crushing weight relief. **(B)** Histogram of quantified GFR data. **(C)** The half-life of FITC-labeled sinistrin in each group of mice. **(D, E)** Measurement of SCr and BUN levels in mouse serum. **(F, G)** qPCR determination of *Kim-1* and *Ngal* mRNA levels in mouse kidney tissues. **(H)** Representative images of HE staining of mice in each group. The arrows represent the site where the injury occurred, such as renal tubular epithelial cell detachment, death, and tubular dilatation. Scar bar: 100 μm *p < 0.05, **p < 0.01, ***p < 0.001. There were six mice in each group. Each scatter represented a mouse sample. All samples were assayed when GFR, SCr, and BUN were performed (n = 6). Three mouse kidney groups were randomly selected for HE staining, and the other 3 mouse kidney tissues were used for RNA extraction to determine *Kim-1* mRNA and *Ngal* mRNA (n = 3).

At first, we evaluated the impact of different crushing weights on kidney function in mice CS-AKI models. The muscle-rich parts of mice’s hind legs were applied with weights of 0.5 kg, 1.0 kg, and 1.5 kg (as shown in Figures 1B, C), equivalent to 25, 50, and 75 times the mice’s body weights. After crushing for 12 h and reperfusion for 6 h, mice were sacrificed to evaluate the biomarkers of renal functions as well as perform pathological staining. Compared with the NC group, the GFR of mice in the different crushing weight groups significantly decreased (as shown in Figures 2A–C). The contents of SCr and BUN in the serum and the mRNA level of kidney *Kim-1* and *Ngal* were elevated in a strength-dependent manner with crushing (Figures 2D–G). HE staining (Figure 2H) further suggested that different crushing weights caused damage to the kidney. As marked by arrows in Figure 2H, detachment of the brush border of the renal tubule, detachment of the tubular epithelial cells, and protein tubular pattern were visible.

### Effect of different crushing duration on kidney function in CS-AKI mice

**FIGURE 3:**
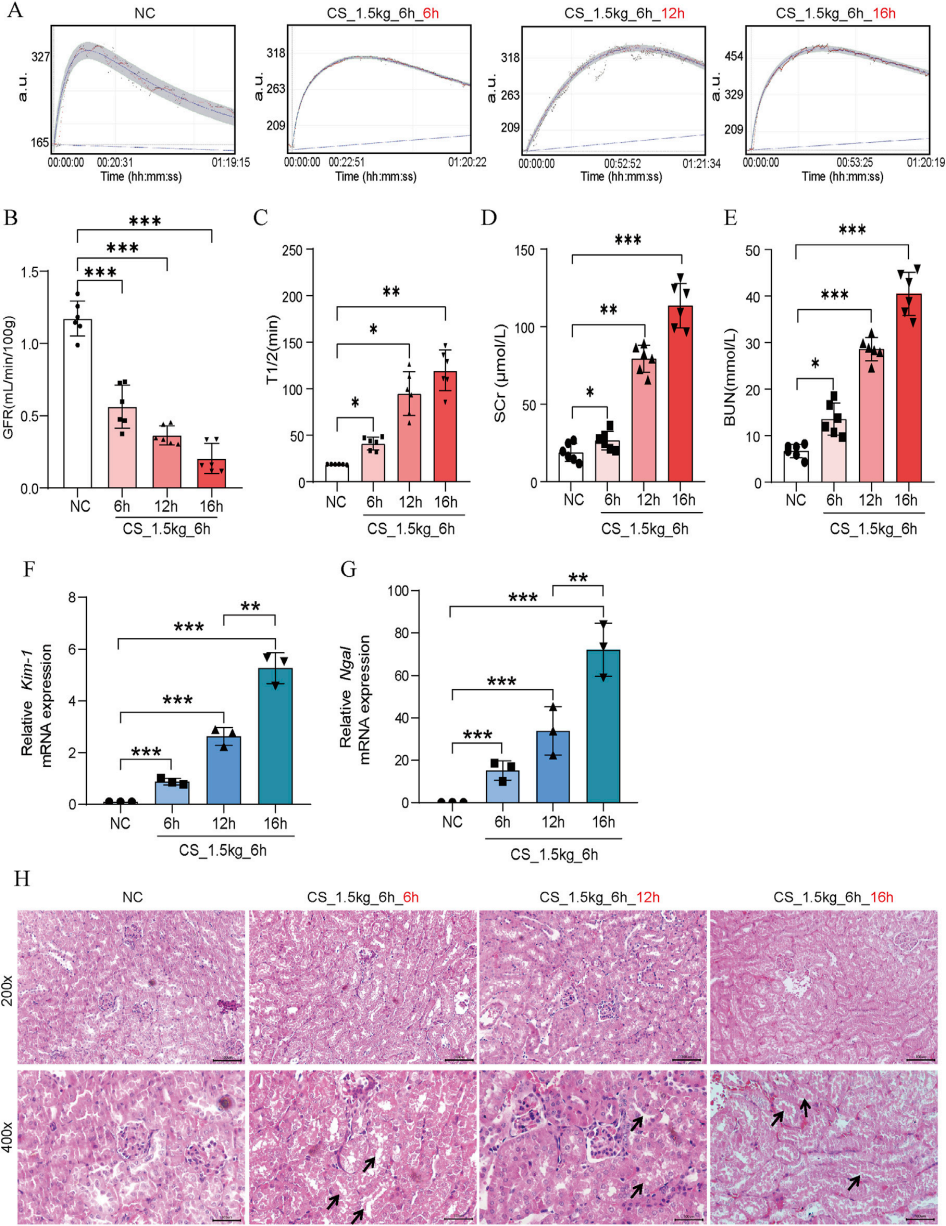
Effect of crushing duration on kidney function in CS-AKI mice. For the crushing duration test: fixed crushing weight of 1.5 kg, decompression time of 6 h. The duration of the applied crushing weight was 6 h, 12 h, and 16 h, respectively. **(A)** The GFR of mice were subjected to a 1.5 kg crushing weight for 6 h, 12 h and 16 h, and measured at 6 h after the crushing weight was relieved. **(B)** Histogram of quantified GFR data. **(C)** The half-life of FITC-labeled sinistrin in each group of mice. **(D, E)** Measurement of serum SCr and BUN levels in mice. **(F, G)** qPCR determination of *Kim-1* and *Ngal* mRNA levels in mouse kidney tissues. **(H)** Representative graphs of HE staining of mice in each group. The arrows represent the site where the injury occurred, such as renal tubular epithelial cell detachment, death, and tubular dilatation. Scar bar: 100 μm *p < 0.05, **p < 0.01, ***p < 0.001.

To investigate the relationship between crushing duration and AKI, we placed mice under heavy weights and crushed them for 6 h, 12 h, and 16 h. The survival rate of mice crushed for more than 16 h was less than 30%, which made it difficult to carry out the follow-up study, so the maximal crushing duration in this study was 16 h. From Figures 3A–C, we can see that the kidney function of the mice decreased significantly in the 6 h after the crushing weight was relieved. The longer the crushing time, the lower the GFR, indicating a severe loss of kidney function in mice. Similar blood SCr and BUN results were also observed (Figures 3D, E). *Kim-1* and *Ngal* mRNA levels also showed time-dependent characteristics (Figures 3F, G). In HE staining (Figure 3H), we found that the kidney tubules showed different degrees of damage, such as tubular expansion, detachment of tubular epithelial cells, and cast formation of tubular in different extrusion time groups. Notably, we found that the longer the crushing durations, the more pronounced the inflammatory cell infiltration.

### Effects of different times of decompression on kidney function in CS-AKI mice

**FIGURE 4:**
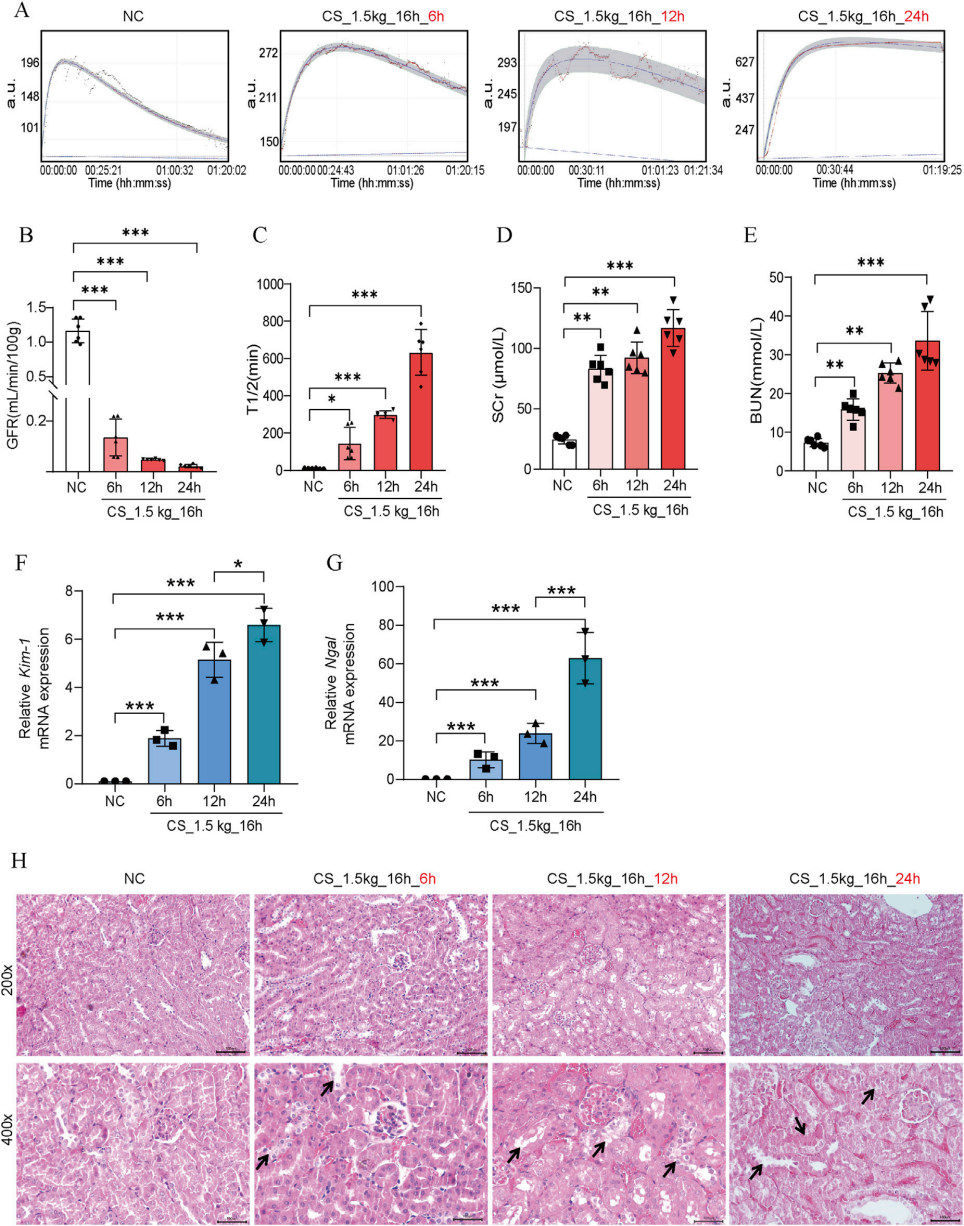
Effect of reperfusion time on kidney function in CS-AKI mice. For the decompression time test: fixed crushing weight of 1.5 kg, crushing duration of 16 h. The relevant indicators of mice were collected at 6h, 12h, and 24 h after the decompression, respectively. **(A)** The GFR was measured at 6 h, 12 h, and 24 h after crushing weight relief in mice pressed at 1.5 kg for 16 h. **(B)** Histogram of quantified GFR data. **(C)** The half-life of FITC-labeled sinistrin in each group of mice. **(D, E)** Measurement of SCr and BUN levels in mice serum. **(F, G)** qPCR determination of *Kim-1* and *Ngal* mRNA levels in mouse kidney tissues. **(H)** Representative graphs of HE staining of mice in each group. The arrows represent the site where the injury occurred, such as renal tubular epithelial cell detachment, death, and tubular dilatation. Scar bar: 100 μm *p < 0.05, **p < 0.01, ***p < 0.001.

It has been shown that within 3–24 h of reperfusion after crushing weight relief in CS mice, the KIM-1 and NGAL protein levels increased significantly, suggesting the occurrence of AKI (Kadioglu et al., 2021). To further investigate the relationship between the time of reperfusion and the severity of AKI, we performed GFR, biochemical and pathologic measurements at 6 h, 12 h, and 24 h after crushing weight relief. As shown in Figures 4A–C, there was a significant decrease in GFR at 6 h of reperfusion, with almost complete loss at 12 h and 24 h. The elevation of SCr and BUN showed a time-dependent pattern, reaching a maximum at 24 h (Figures 4D, E). The levels of *Kim-1* mRNA and *Ngal* mRNA also showed a rapid increase (Figures 4F, G). In HE staining (Figure 4H), we found that tubular dilatation was more significant in the model group, especially at 24 h. This might be related to the damage of oxygen free radicals and inflammatory factors to renal tubular epithelial cells after reperfusion.

### Glycerol-induced RM-AKI

**FIGURE 5:**
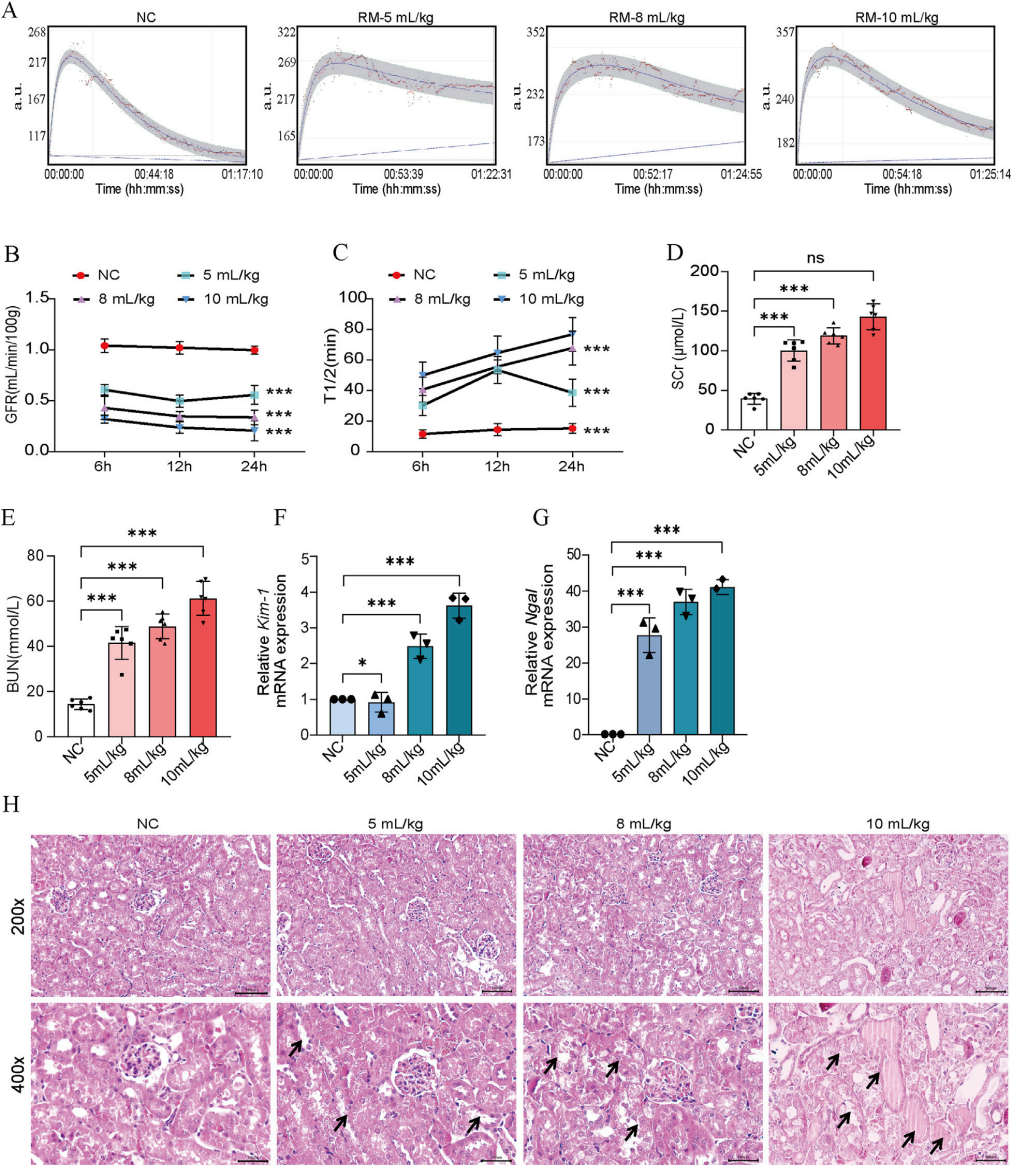
50% glycerol induced RM-AKI. **(A)** Determination of GFR in mice 24 h after different 50% glycerol injection doses (5 mL/kg, 8 mL/kg, 10 mL/kg). **(B)** Histogram of quantified GFR data. **(C)** The half-life of FITC-labeled sinistrin in each group of mice. **(D, E)** Measurement of serum SCr and BUN levels in mice. **(F, G)** qPCR determination of *Kim-1* and *Ngal* mRNA levels in mouse kidney tissues. **(H)** Representative graphs of HE staining of mice in each group. The arrows represent the site where the injury occurred, such as renal tubular epithelial cell detachment, death, and tubular dilatation. Scar bar: 100 μm *p < 0.05, **p < 0.01, ***p < 0.001.

We found that RM-AKI mice presented significant AKI features, with decreased GFR, elevated levels of SCr, BUN, *Kim-1* mRNA, and *Ngal* mRNA (Figures 5A–G). This is consistent with previous studies (Afolabi et al., 2023). HE staining showed that the pathologic changes of RM-induced AKI presented more pronounced renal tubular epithelial cell detachment, death, and tubular dilatation (Figure 5H). These data suggested that RM-AKI might be an ideal candidate model for CS-AKI. A significant protein cast was presented in the high-dose glycerol injection group (10 mL/kg) (Figure 5H).

## Discussion

In this study, we investigated the effects of different crushing weights, time, and decompression time on kidney function in mice to rationalize the establishment of CS-AKI mouse models. Although these factors are difficult to control in real trauma scenarios, they are important for understanding the mechanism of CS-AKI and for optimizing clinical management, developing therapeutic regimens, and improving prognosis.

The mortality rate at disaster sites is high, and survivors rescued from the rubble commonly suffer from CS (Erek et al., 2002). Patients with CS typically present hyperkalemia, RM, myoglobinuria, and severe blood loss (Ramirez-Guerrero et al., 2024). Thus, AKI may be caused by multiple factors, including RM, kidney ischemia, and myoglobin accumulation (Gibney et al., 2014). Myoglobin is a negatively charged molecule with a molecular weight of 17 KD and is found in large quantities in rhabdomyosin (skeletal and cardiac muscle) cells. Previous studies have shown that post-traumatic RM releases of large amounts of myoglobin from destroyed skeletal muscle into circulation (Westphal, 2019). Due to the small molecular weight of the myoglobin, it can pass through the primary membrane aperture of the glomerular filtration barrier (∼69 KD). However, a large amount of myoglobin flowed through the kidneys within a short period and accumulated in the tubules to block the renal tubules. Moreover, it is straightforward to interact with the Tamm-Horsfall protein in the renal tubules in an acidic environment and precipitate to form myoglobin tubular cast, which clogs up the tubular and impairs kidney function (Long et al., 2019). Our study found that crushing durations and crushing weight were crucial for the formation of tubular myoglobin, which became progressively more numerous with the prolongation of crushing durations and increased crushing weight. This may be because the deepening of the squeeze time and crushing weight caused more severe RM, and therefore, much myoglobin was released and accumulated in the kidney, exacerbating myoglobin tubular forms.

Due to the unpredictability of events such as disasters and automobile accidents, it is difficult to provide adequate preventive protection for patients with CS. Moreover, from previous seismic data, the severity of CS suffered by patients is not consistent due to the different scenarios of disasters, which is highly correlated with the time of being buried by the debris (crushing durations) and the strength of being crushed (Pan et al., 2019). It is easily understood that crushing durations (rescue time) affect patient prognosis. In a retrospective study on rescue time and post-earthquake mortality, scholars found that rescue time was strongly correlated with mortality, with the optimal rescue time being 12 h for children and 24 h for adults (Pan et al., 2019). In this study, we explored crushing weight and crushing durations. We used 0.5 kg, 1.0 kg, and 1.5 kg weights to crush on the muscle area of both legs of mice, equivalent to 25, 50, and 75 times their body weight. The results showed that with increasing weight, the mice showed a significant decrease in GFR and an increase in SCr and BUN, suggesting an increased degree of kidney function impairment, which correlated with the severity of clinical AKI. *Kim-1* and *Ngal* mRNAs are considered to be sensitive and specific markers of renal tubular injury (Chen et al., 2023). In the present study, they were also shown to increase dramatically in the kidneys of AKI mice. The levels of SCr, BUN, *Kim-1* mRNA, *Ngal* mRNA, and GFR were used as effective markers of AKI to comprehensively evaluate the severity of kidney injury in CS or RM mice from biochemical, molecular, and renal function perspectives, respectively. It has been suggested that a negative hyperbolic relationship between SCr and GFR, with a 50% increase in SCr, potentially translates into a decrease in GFR of approximately 25% (Stevens et al., 2006). Direct GFR measurement is traditionally the gold standard of kidney function assessment (Zhang et al., 2021). In our research, GFR assessment provides a good way to distinguish different groups for renal function. Tissue damage may be more severe under high crushing weight, induced by cell death, further aggravating kidney injury (Teksen et al., 2019). The decline in kidney function was more pronounced in mice with prolonged crushing durations, suggesting that the time factor significantly impacts the prognosis. It was reported that kidney cortical blood flow was significantly reduced during CS. Reperfusion triggered inflammatory events (Murata et al., 2016). This is consistent with the observation in this study that the longer the duration of the crush, the more severe the renal inflammatory cell infiltration. In terms of decompression time exploration, the degree of kidney injury in mice increased continuously with longer decompression time, indicating irreversible damage after reperfusion, which may be associated with a sustained elevation of intra- and extracellular free radicals and inflammatory state secondary to decompression (Okubo et al., 2023).

Glycerol injection-induced RM is considered an ideal alternative model of CS due to its similar pathogenesis (Taguchi et al., 2020). 50% glycerol (dose: 5–10 mL/kg) is commonly used to construct a mouse model of RM because of the pronounced damaging effects of glycerol on muscle (Liu et al., 2019). It was reported that myoglobinuria, plasma creatine kinase, plasma hemoglobin, kidney tissue iron, and inflammation levels were significantly higher than those in the control group at 5 h after glycerol injection (Yuqiang et al., 2022; Williams et al., 2023). In order to investigate the connection and difference between CS-AKI and RM-AKI, we constructed an RM-AKI model using three different glycerol doses of 5, 8, and 10 mL/kg. The RM mice presented significant kidney injury features such as decreased GFR and elevated serum SCr and BUN. These findings are consistent with previous studies (Sun et al., 2022). CS-AKI and RM-AKI models had comparable SCr and BUN level values. However, the kidney tissue *Kim-1* mRNA and *Ngal* mRNA levels in RM-AKI mice were not as high as those in CS-AKI mice. And the high dose of glycerol injection caused protein casts. This implies that the results of the two modeling methods are different at the molecular pathology level. Overall, these data support the idea that RM-AKI could be an ideal candidate model for CS-AKI.

Our study has some limitations. This study did not perform an in-depth mechanistic study to explore the causes of AKI occurrence, and whether CS-AKI is associated with programmed cell death, inflammation, and oxidative stress levels was not well described. To avoid the complexity that changes in estrogen may introduce to modeling CS mice (Yu et al., 2019), all mice used in this study were male, which is consistent with previous studies (Yang et al., 2020; Lv et al., 2021). Although our study provides important data for the field, the choice of animal sex may limit the generalizability of the results. Future studies should consider gender as a variable for a more comprehensive understanding of the pathological mechanism of CS.

## Conclusion

In conclusion, we designed a multi-channel small animal crushing platform and constructed a mouse CS-AKI model under different crush conditions by adjusting the crush strength of the higher, the longer, and the time of reperfusion after decompression, and evaluated the kidney function of the model. The gold standard GFR of the kidney function assessment contributes to the model building. The relevant experimental parameters provide a reference for the future construction of CS-AKI studies with different severities. Our study suggests that oxidative stress and inflammation may play an important role in the pathogenesis of CS-AKI. It also implies that patients who are subjected to more robust and longer compression and longer reperfusion time after decompression face a more dangerous situation and that treatment protocols should take into account early control of inflammation as well as myoglobin levels. The results of this study also provide clues to investigate the mechanisms of traumatic kidney injury and optimize prognostic management.

## Data Availability

The original contributions presented in the study are included in the article/supplementary material, further inquiries can be directed to the corresponding authors.
